# Life on the dry side: a roadmap to understanding desiccation tolerance and accelerating translational applications

**DOI:** 10.1038/s41467-025-58656-y

**Published:** 2025-04-06

**Authors:** R. A. Marks, J. T. B. Ekwealor, M. A. S. Artur, L. Bondi, T. C. Boothby, O. M. S. Carmo, D. C. Centeno, K. K. Coe, H. J. W. Dace, S. Field, A. Hutt, S. Porembski, A. Thalhammer, L. van der Pas, A. J. Wood, P. Alpert, D. Bartels, S. Boeynaems, M. N. Datar, T. Giese, W. I. Seidou, S. M. Kirchner, J. Köhler, U. G. V. S. S. Kumara, J. Kyung, R. Lyall, B. D. Mishler, J. B. V. T. Ndongmo, M. S. Otegui, V. Reddy, J. Rexroth, S. M. Tebele, R. VanBuren, J. Verdier, U. C. Vothknecht, M. F. Wittenberg, E. Zokov, M. J. Oliver, S. Y. Rhee

**Affiliations:** 1 Plant Resilience Institute, Michigan State University, East Lansing, MI USA; 2 Department of Plant Biology, University of Illinois, Urbana, IL USA; 3 Department of Biology, San Francisco State University, San Francisco, CA USA; 4 Laboratory of Plant Physiology, Wageningen Seed Science Centre, Wageningen University, Wageningen, The Netherlands; 5 Department of Botany, University of Rostock, Institute of Biosciences, Rostock, Germany; 6 Department of Molecular Biology, University of Wyoming, Laramie, WY USA; 7 Department of Molecular and Human Genetics, Baylor College of Medicine, Houston, TX USA; 8 Universidade Federal do ABC, São Bernardo do Campo, Brazil; 9 Department of Biology, Middlebury College, Middlebury, VT USA; 10 Delft University of Technology, Delft, The Netherlands; 11 Department of Biochemistry and Molecular Biology, Michigan State University, East Lansing, MI USA; 12 Department of Plant Biology, Michigan State University, East Lansing, MI USA; 13 Department of Plant, Soil, and Microbial Sciences, Michigan State University, East Lansing, MI USA; 14 University of Texas at Austin, Austin, TX USA; 15 Department of Physical Biochemistry, University of Potsdam, Potsdam, Germany; 16 Department of Molecular and Cell Biology, University of Cape Town, Cape Town, South Africa; 17 School of Biological Sciences, Southern Illinois University, Carbondale, IL USA; 18 University of Massachusetts-Amherst, Amherst, MA USA; 19 Department of Integrative Biology, University of California at Berkeley, Berkeley, CA USA; 20 IMBIO, University of Bonn, Bonn, Germany; 21 Therapeutic Innovation Center (THINC), Baylor College of Medicine, Houston, TX USA; 22 Center for Alzheimer’s and Neurodegenerative Diseases (CAND), Texas Children’s Hospital, Houston, TX USA; 23 Dan L Duncan Comprehensive Cancer Center (DLDCCC), Baylor College of Medicine, Houston, TX USA; 24 Jan and Dan Duncan Neurological Research Institute, Texas Children’s Hospital, Houston, TX USA; 25 Agharkar Research Institute, Pune, India; 26 WASCAL, Universite Felix Houphouet-Boigny, Abidjan, Côte d’Ivoire; 27 Center of Plant Systems Biology and Biotechnology, Plovdiv, Bulgaria; 28 Department of Integrative Biology, University and Jepson Herbaria, University of California, Berkeley, CA USA; 29 University of Wisconsin-Madison, Madison, WI USA; 30 Botanic Gardens, Tissue Culture Laboratory, Parks Recreation and Culture Unit, eThekwini Municipality, Durban, South Africa; 31 Forest Ecology and Management Department, Swedish University of Agricultural Sciences, Uppsala, Sweden; 32 Univ Angers, Institut Agro, INRAE, IRHS, SFR QUASAV, Angers, France; 33 Institute of Cellular and Molecular Botany, University of Bonn, Bonn, Germany; 34 Division of Plant Sciences and Technology, University of Missouri, Interdisciplinary Plant Group, Columbia, MO USA

**Keywords:** Ecology, Evolution, Biophysics, Systems biology, Computational biology and bioinformatics

## Abstract

To thrive in extreme conditions, organisms have evolved a diverse arsenal of adaptations that confer resilience. These species, their traits, and the mechanisms underlying them comprise a valuable resource that can be mined for numerous conceptual insights and applied objectives. One of the most dramatic adaptations to water limitation is desiccation tolerance. Understanding the mechanisms underlying desiccation tolerance has important potential implications for medicine, biotechnology, agriculture, and conservation. However, progress has been hindered by a lack of standardization across sub-disciplines, complicating the integration of data and slowing the translation of basic discoveries into practical applications. Here, we synthesize current knowledge on desiccation tolerance across evolutionary, ecological, physiological, and cellular scales to provide a roadmap for advancing desiccation tolerance research. We also address critical gaps and technical roadblocks, highlighting the need for standardized experimental practices, improved taxonomic sampling, and the development of new tools for studying biology in a dry state. We hope that this perspective can serve as a roadmap to accelerating research breakthroughs and unlocking the potential of desiccation tolerance to address global challenges related to climate change, food security, and health.

## Abstract (web summary)

Desiccation tolerance is an extreme adaptation to water limitation. Here the authors envisage a roadmap to advance desiccation tolerance research, highlight key knowledge gaps, call for standardization, and explore potential applications in conservation, medicine, agriculture, and biotechnology.

## Introduction

Desiccation tolerance is one of nature’s most extraordinary phenomena. Macromolecules, cells, and organisms typically require high internal hydration to function^1^ and most of our understanding of biology occurs within a narrow moisture window. However, there are some organisms, tissues, and cells that can survive the near complete loss of internal water without dying. Desiccation-tolerant cells and organisms survive drying so extreme that there is insufficient liquid water to form even a single layer of hydration around cellular structures and molecules^2–4^. Understanding the adaptive mechanisms that preserve life in a desiccated state holds promise for various practical applications, including the production, storage, and utilization of agricultural, medicinal, and material products. For example, insight into this phenomenon could drive innovations in optimizing dry storage of germplasms and labile macromolecules, facilitating the long-term preservation of natural diversity, and has potential to accelerate the bioengineering of more resilient crops. Advancing these objectives is critical–now more than ever–given the unprecedented rates of species and genetic diversity loss and the increasing frequency and magnitude of natural disasters^5^.

**Fig. 1:**
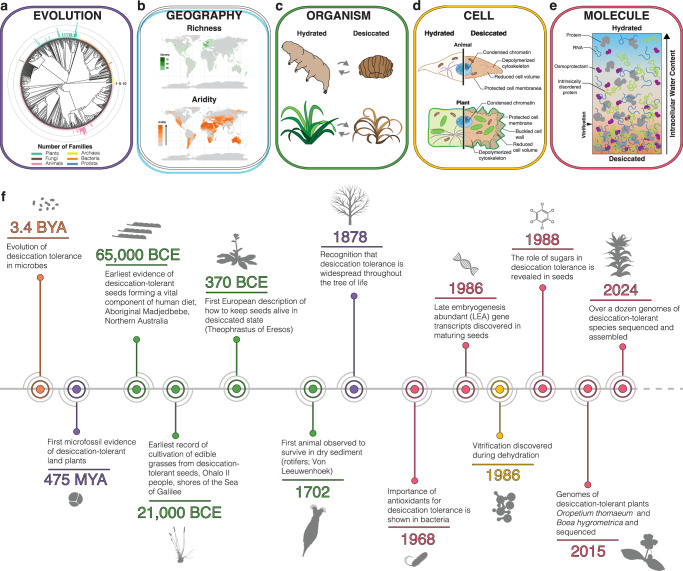
Overview of desiccation tolerance research. Summaries of **a** evolutionary, **b** ecological, **c** morphological, **d** cellular, and **e** molecular aspects of desiccation tolerance. **f** timeline of major natural and research milestones in desiccation tolerance. Colors represent the biological scale of each discovery: purple for discoveries in evolution, blue for discoveries in geography, green for organismal-scale discoveries, yellow for cellular-scale discoveries, and red for discoveries on the molecular scale. Orange represents a historical biological event. For a brief history of the modern discovery of desiccation tolerance, see Alpert (2000). Illustrations in (**c**–**e**) by Rachel Torrez.

The phenomenon of desiccation tolerance has long captivated and perplexed scientists (Fig. 1)^6–17^, but unraveling the evolutionary, physiological, and genetic basis of this trait has proven a formidable challenge. The inherent complexity of desiccation tolerance and diversity of desiccation-tolerant organisms, coupled with the technical limitations in experimental assays that require an aqueous environment, has hindered progress. Still, research on desiccation tolerance has accelerated in recent years^18,19^, and this growing interest, combined with technological advances in omics and other high-throughput methodologies, has led to a wealth of data across numerous modalities. This growth offers exciting opportunities to accelerate breakthroughs and potential translational applications, but integration across diverse study systems, scales of biological organization, and individual labs remains a roadblock. Historically, desiccation tolerance research has been conducted in siloed sub-disciplines defined primarily by study organisms, with limited integration of findings across kingdoms^18^. Currently, there is still considerable variation in the organisms and questions being investigated, as well as the methodologies employed and outcomes tracked. While this diversity is an asset for data and hypothesis generation, standardization across sub-disciplines is necessary to facilitate integration and unlock the full potential of desiccation tolerance research.

Here, we provide an overview of the current understanding of desiccation tolerance, summarize gaps and technical roadblocks that should be addressed to accelerate desiccation tolerance research, and highlight its potential translational applications. We outline core conceptual frameworks and methodologies central to desiccation tolerance research, and present working definitions of key terms. We suggest generalized best practices that span organisms and sub-disciplines, which we hope will facilitate knowledge integration. As the field becomes more unified through the adoption of these shared practices, we anticipate significant advances in xeropreservation technology, synthetic biology, and other futuristic applications.

### Definition of desiccation tolerance

What is desiccation tolerance? And what does it mean to survive or maintain viability in a dry state? Developing language and consensus around these concepts is critical for integration across the field. We propose a set of working definitions (Box 1), built on historical frameworks, which could be readily adopted by diverse sub-disciplines to facilitate collaboration. To contextualize these definitions, it is important to note that desiccation tolerance and the related term ‘anhydrobiosis’ are fundamentally different from drought avoidance and resistance. Desiccation-tolerant cells dehydrate so completely that essentially all measurable cellular activity ceases, yet somehow they resume healthy cellular function within minutes to hours of rehydration. Desiccation-tolerant cells possess adaptations, either constitutive or induced during dehydration, which preserve cellular integrity in a dry state and support repair upon rehydration. Decades ago, desiccation tolerance was defined as the ability to revive from equilibration with the water potential of the air, which is predominantly low^2^. In practice, this often equated to surviving equilibration to 50% relative humidity at 20 °C, which corresponds roughly to a water potential of −100 MPa and the point at which the monolayer of water molecules around cellular organelles breaks down^4,20^. Similarly, anhydrobiosis was defined as the process by which an organism can maintain viability in the dried state where there is insufficient water to support the monolayered sphere of hydration around macromolecules and membranes essential for enzyme activity^3,21,22^. These definitions, though useful, lack flexibility regarding intermediate water potentials where biological activity is severely inhibited, despite residual water, and do not fully account for the multiple physiological, ecological, and anatomical factors that influence desiccation tolerance. Therefore, we suggest updated definitions (Box 1) that acknowledge the spectrum of dehydration, placing desiccation at one extreme and hydration at the other. While we still find it useful to define thresholds and cutoff points highlighting the extremity of desiccation tolerance, we suggest that tolerance should be described on a continuum, allowing for a more quantitative assessment. Massive shifts in cellular dynamics and material properties occur between −5 MPa and −100 MPa^20,23^, and tolerance of these intermediate dehydration states is notably variable across organisms and tissues. Dehydration tolerance should therefore be described in relation to minimum recorded water content of the sample, with desiccation tolerance being reserved only for those samples with a minimum recorded water potential < −100 MPa (Box 1).

Box 1 Working definitions **Anhydrobiosis:** The process of drying to a quiescent state, where there is insufficient water to hydrate cellular macromolecules and membranes, and resuming normal cellular function when rehydrated.

**Desiccation tolerance:** The ability to dry to a quiescent state and resume normal cellular function when rehydrated. Typically, organisms and cells are considered desiccation-tolerant when they can revive after being dried to, or below, −100 MPa (~10% dry mass).

**Dehydration tolerance:** The ability to dry to cellular water potentials between −5 MPa and −100 MPa and resume normal cellular function when rehydrated. In practice, this should be described in relation to the minimum recorded water content of a sample.

**Dehydration sensitivity:** Inability to maintain viability after cellular water potential drops below −5 MPa

**Maintain viability:** Preservation of cellular integrity, such that normal cellular function, including metabolism, growth, and reproduction resume when cells regain full turgor.
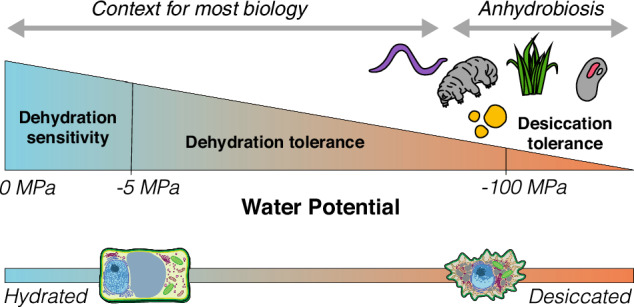

### Evolution of desiccation tolerance

Understanding the evolution of desiccation tolerance is critical for identifying the genetic, physiological, and ecological conditions that enable life at the extreme. The evolutionary processes that gave rise to desiccation tolerance also provide a blueprint for potential translation into applied contexts. Desiccation tolerance has evolved recurrently and convergently across the tree of life^24–27^ and is widely distributed across taxa, spanning diverse prokaryotic and eukaryotic lineages (Fig. 1a). Desiccation tolerance is an ancestral adaptation to periodically dry conditions that played a critical role in enabling the transition of early life from aquatic to terrestrial environments. Desiccation tolerance likely arose in ancestral bacteria, archaea, and algal populations as a response to periodic drying^28^. The rise and diversification of terrestrial organisms was enabled by ancestral traits present in these lineages^29–32^. As some lineages diversified and adapted to terrestrial life, they evolved other mechanisms to cope with water scarcity, and many lineages lost their ability to tolerate desiccation as they evolved alternative ways to escape or resist drought^33^. Interestingly, a few animals maintained desiccation tolerance in their eggs or larvae, as did many vascular plants in their seeds or spores, indicating that the genetic potential for desiccation tolerance was widely retained across hundreds of millions of years of evolution, but its expression was often developmentally restricted to specific tissue types and life stages. In vascular plants, the occurrence of desiccation tolerance in vegetative tissues is thought to be secondary, as compared to the ancestral vegetative desiccation tolerance present in extant streptophytes and bryophytes. This secondary evolution is thought to have occurred through the rewiring of ancestral pathways that were maintained in spores and seeds^34–36^.

There have been multiple evolutionary losses of desiccation tolerance, suggesting that there is a cost associated with maintaining desiccation tolerance when it is not required for survival^29,33,37^. Indeed, tradeoffs do exist between desiccation tolerance and other traits, such as growth and productivity^33^, and these tradeoffs partially explain the restricted distribution of many desiccation-tolerant organisms, although there are exceptions^38^. The evolution of desiccation tolerance is often associated with expansion into marginal habitats, such as bare rock outcrops in the tropics^39^, tidal zones^40^, and hyper-arid microclimates. Desiccation tolerance has evolved, been lost, and sometimes subsequently re-gained at different times than the genes that influence desiccation tolerance phenotypes, complicating the identification of evolutionary homologies. For example, genes that underlie non-homologous desiccation tolerance phenotypes (e.g., due to convergent evolution) might well be homologous (e.g., due to shared ancestry), highlighting that some lineages could be genetically predisposed to evolve desiccation tolerance.

The complex evolutionary history of desiccation tolerance has given rise to a wide diversity of desiccation-tolerant organisms and tissues. Not surprisingly, there is substantial variability in the combination of traits expressed in different lineages, and this remains poorly understood, especially on a broad phylogenetic scale^41^. However, comparisons within and across taxa have been leveraged to identify phenotypic and genomic homologies underlying desiccation tolerance^27,42–46^. For example, a growing number of sister-species comparisons in vascular plants within families such as *Selaginellaceae*, *Linderniaceae*, and *Poaceae* have shown that the convergent expansion of Early Light Inducible Proteins (ELIPs) distinguish the genomes of desiccation-tolerant plants^47–53^. Desiccation tolerance also appears to build on the deeply conserved genetic architecture of water deficit responses^27,42,46^ and similar genetic features are evident across diverse taxa^43^. Comparative studies at increasingly shallow evolutionary time scales (e.g., within single species) have highlighted the role of gene duplication and polyploidy in enhancing desiccation tolerance^45^. Phenotypic plasticity in desiccation tolerance also exists, perhaps best studied in bryophytes^54,55^ and is itself a trait that can itself evolve. Phenotypic plasticity is a matter of degree, so rather than using categorical terms, a more quantitative framework such as the norm of reaction approach, which compares the phenotypes of each genotype across an environmental gradient^56^ is recommended. Untangling the complex evolutionary dynamics of desiccation tolerance requires integration across scales and study systems.

Technological and computational advancements have accelerated the use of omics approaches for untangling the evolutionary history of desiccation tolerance across kingdoms^57,58^. Currently, over a dozen genome assemblies of desiccation-tolerant or so-called “resurrection” plants have been published including for *Boea hygrometric*a^16^, *Craterostigma plantagineum*^59^*, Eragrostis nindensis*^52^, *Haberlea rhodopensis*^60^*, Lindernia breviden*s^49^, *Microchloa afra*^27^*, Oropetium thomaeum*^15,61^*, O. capense*^27^*, Selaginella tamariscina, S. lepidophylla*^50^*, Sporobolus stapfianus*^53^*, Syntrichia caninervis*^17^*, S. ruralis, Tripogon minimus*^27^*, and Xerophyta schlechteri*, many of which are hosted at the Drying Without Dying database (http://desiccation.novogene.com/home)^62^ and are coupled with vast RNAseq and multi-omics datasets. Comparatively, fewer genomic resources are available for desiccation-tolerant animals, but genome assemblies exist for the midge *Polypedilum vanderplanki*^63^, tardigrades *Ramazzottius varieornatus*^64^ *Hypsibius dujardini*^65^ *and Paramacrobiotus sp*^66^, brine shrimp *Artemia franciscana*^67^, rotifers *Adineta vaga*^68^, *Rotaria macrura*, and *R*. *magnacalcarata*^69^, and nematodes *Aphelenchus avenae*^70^, *Anguina tritici*^71^, and *Caenorhabditis elegans*^72^. Genome assemblies are also available for numerous desiccation-tolerant algae^73^, bacteria, and fungi. Comparative studies that leverage these phylogenetically diverse datasets are a powerful way to understand the evolutionary history and mechanisms of desiccation tolerance^43^. However, widespread gaps in taxonomic sampling and genomic resources hinder our ability to reconstruct the deep evolutionary history of desiccation tolerance, and additional data are needed to understand contemporary evolution and local adaptation.

### Ecology of desiccation tolerance

Understanding the ecological dynamics of desiccation-tolerant species is critical for identifying processes that sustain ecosystems and communities in extreme environments. This knowledge will provide a foundation for translating natural processes into conservation efforts, sustainable management practices, and ecosystem engineering. In many cases, desiccation-tolerant organisms can tolerate a variety of extreme conditions beyond water limitation, including temperature extremes above 100 °C and below 0 °C, high salinity, nearly complete vacuum, intense radiation, and toxins^2,74,75^. This remarkable cross-tolerance is typically only observed in the desiccated state^76,77^, but many species also have mechanisms for accelerating their life cycle^78–80^ and avoiding carbon starvation^81,82^ that facilitate survival in extreme environments.

It might seem intuitive that desiccation-tolerant organisms would be linked to arid regions, but this is common only in some taxonomic groups^24,83^. Many desiccation-tolerant organisms are found in wetter climates but in microhabitats where water availability is sometimes or always very low, including hypersaline lakes, ephemeral pools, rock outcrops, or tree trunks and canopies^76,84,85^. So while desiccation-tolerant organisms can be found almost anywhere on Earth, taxa are not evenly distributed (Fig. 1b). Some groups have a cosmopolitan distribution such as desiccation-tolerant tardigrades, nematodes, lichens, bryophytes, and seeds, which are all found from the tropics to Antarctica^83,86^, but other groups show more geographical and ecological specificity. For example, the diversity of angiosperms with vegetative desiccation tolerance increases towards the tropics and in areas with moderate seasonal conditions^41,84^. More subtle patterns of spatial distribution and habitat specification are evident within narrower taxonomic groups. For example, desiccation-tolerant eudicots are nearly absent from the Americas^41^. It is likely that inter- and intra-specific differences in desiccation tolerance phenotypes partially explain ecological and biogeographical patterns. Species native to drier environments tend to tolerate more rapid, complete, or prolonged desiccation^81^ than species from more mesic environments.

There is a need to link species responses to community dynamics and ecosystem functions, particularly in heterogeneous environments where multiple selective pressures are at play. While data in this area remain limited, assessing community-level dynamics of desiccation tolerance has been explored in some systems. For example, soil crusts that combine desiccation-tolerant bacteria, fungi, algae, lichens, and bryophytes decrease erosion and fix nitrogen in arid systems worldwide and are strongly affected by both climate change and human disturbance^87,88^. Investigating the interaction of soil crusts (and the desiccation-tolerant organisms that comprise them) with other ecological processes will enhance our understanding of the roles of desiccation tolerance in maintaining biodiversity, ecosystem resilience, and stability.

### Physiology and cell biology of desiccation tolerance

Understanding the precise physiological and cellular mechanisms that enable desiccation tolerance is essential for nearly all potential applications. Desiccation tolerance is a complex, emergent phenotype that requires the coordination of numerous cellular processes and biochemical pathways. While many of the central cellular processes are known, how they are regulated and coordinated remains unclear. Despite the diversity of desiccation-tolerant organisms, certain responses are widely shared across distantly related taxa^20,43^. For example, leaf curling is commonly observed in desiccating plants (Fig. 1c)^53,89–93^. Similarly, the dauer larvae of midges and nematodes coil up, and tardigrades contract their bodies and retract their limbs during desiccation (Fig. 1c)^25,86,94^. These structural changes could simply be a consequence of water loss but are also thought to help mitigate mechanical damage due to volume loss, protect brittle appendages, and reduce exposure to photooxidative damage. The pre-emptive cessation of metabolism, including photosynthesis in photoautotrophs, also occurs during desiccation. However the cessation of metabolic activity is a consequence of dehydration^95^ that does not predict survival, but is only reversible in tolerant cells upon rehydration.

The impacts of dehydration and desiccation on cellular macromolecules and compartments are multifacteted: from intracellular molecular crowding and concomitant higher local concentrations of damaging reactive oxygen species (ROS), to the loss of the molecular hydration layer^94,96^. ROS-associated DNA damage accrues during drying and in the dry state^97–99^ but chromatin condensation observed in desiccation-tolerant tissues may minimize damage (Fig. 1d)^98,100–105^. Increased molecular concentration leads to partial protein unfolding, misfolding, and aggregation^106^, which in turn affects the function of enzymes and macromolecular complexes, with the electron transport chains of chloroplasts and mitochondria being major sites of damage in sensitive organisms^107,108^. In desiccation-tolerant plants and some bacteria, gross changes in cell wall shape are observed, and these dynamics require cellular and tissue remodeling (Fig. 1d)^109–111^. Cellular integrity is maintained under these conditions by complex cell wall remodeling and folding to alleviate mechanical stress^112^, or by increased vacuolation to maintain volume and shape^111^. The cytoskeleton also undergoes significant changes as microtubules depolymerize during drying and reassemble during rehydration (Fig. 1d)^113^. Lipid bilayers exhibit increased fusion events and lipid phase transitions (Fig. 1d), which desiccation-tolerant organisms counter by altering lipid composition early in the drying response^114^.

Desiccation-tolerant organisms exhibit a dynamic accumulation of protective proteins during the early phase of drying, including various DNA-binding proteins, late embryogenesis abundant (LEA) proteins, heat shock proteins (HSPs), lipocalins, and, in plants, ELIPs (Fig. 1e)^106,115–119^. Many of these proteins are intrinsically disordered (IDPs) and likely help to prevent protein aggregation, unfolding, and membrane disruption, thereby preserving cellular organization^106,120^. Some IDPs have now convincingly been shown to confer protection via percolation or gel transitions that maintain cellular organization and prevent drying-induced damage^95,121–123^. The formation of membrane-less compartments via biomolecular condensation or gelation of proteins could also play a role in the desiccation tolerance phenotype^124–128^^.^

In desiccation-tolerant organisms, drying also leads to major shifts in carbohydrate metabolism and the accumulation of protective metabolites^129,130^. Non-reducing sugars such as trehalose, raffinose, and sucrose play central roles in stabilizing desiccated cells and tissues across various life forms, including bacteria, yeast, nematodes, invertebrates, desiccation-tolerant (orthodox) seeds, and vegetatively desiccation-tolerant plants^130–133^. For example, animals like brine shrimp, nematodes, and tardigrades accumulate trehalose^25,130,134^, and the accumulation of raffinose or sucrose appears to play an analogous role in many desiccation-tolerant plants^135^. DNA damage is mitigated by DNA-binding proteins that enhance desiccation protection^136^ but there is also evidence of upregulated DNA repair machinery during drying^98^, suggesting that both DNA protection and repair contribute to establishing the maintenance of DNA integrity when the cells reach the desiccated state. Similarly, sugars and IDPs appear to function synergistically to help maintain membrane fluidity^114,130^ during the drying process.

At sufficiently low water content, the cytosol will undergo vitrification to adopt a non-crystalline or “glassy” solid state (Box 1; Fig. 1d, e)^137^. This state reduces molecular motions that would otherwise allow for the unfolding and aggregation of proteins, the fusion of membranes, and the general loss of cellular organization and integrity^115,138^. While cytosol vitrification is associated with desiccation tolerance, it is not sufficient to confer tolerance, as any sufficiently heterogeneous system (e.g., a cell) will form a vitrified, non-crystalline state upon drying^139^. The ‘vitrification hypothesis’ has been a longstanding theory which posits that desiccation-tolerant organisms survive drying by vitrifying or forming non-crystalline glasses. The theory suggests that by forming intracellular glasses, an organism induces a super-viscous state in which molecular motions, such as protein unfolding, are slowed to the point that they no longer take place on biological time scales–thus preserving biological form and function. Current work is focused on identifying the properties that distinguish a protective vitrified system from a non-protective one. While there is clearly much more to learn about the material properties and biophysics underlying protective vitrification, an emerging picture makes it clear that glass transition temperature is not the only property contributing to protection by glasses in the dry state^139–141^. Other properties, such as glass former fragility, a measure of how a system’s viscosity increases as it approaches its glass transition temperature and/or dries, also often trend positively with survival in the dry state^25,139,140,142^.

## Roadblocks and key questions in desiccation tolerance research

Despite significant progress in understanding the mechanisms underlying desiccation tolerance, many questions remain (Box 2), and persistent roadblocks hinder comprehensive insights and translational applications. A critical challenge is the lack of standardization in experimental practices, including different methods of assessing water status, methods of drying and rehydrating specimens, the timing of sampling, set of traits measured, and selection of metadata reported. This variability complicates comparisons and synthesis across different studies, organisms, and scales of biological organization. Technical limitations to studying biology in a dry state, including the reliance on water in traditional cell biology techniques, taxonomic sampling gaps, and sparse ecological data, pose added challenges.

To advance research on desiccation tolerance, answer key questions (Box 2), and accelerate potential translational applications, it is essential to address these gaps through the adoption of standardized practices and by promoting the open and equitable sharing of resources to strengthen collaborations. Below, we present a brief guide to central concepts, approaches, and best practices for data generation, standardization, curation, and synthesis in the context of desiccation tolerance. We outline classic dehydration and rehydration experiments, discuss key measurements, and propose essential metadata that can be used to answer key questions in the field.

Box 2 Key questions in desiccation tolerance research **Evolutionary origins**

- How has desiccation tolerance evolved across different taxa, and what genetic, physiological, and ecological factors influence its emergence and loss?
- What are the evolutionary tradeoffs between desiccation tolerance and other traits, such as growth, metabolic rate, and reproduction?

**Ecological dynamics**

- How does the distribution of desiccation tolerant species correlate with environmental factors?
- What ecological interactions and community-level dynamics support desiccation tolerance?
- How do desiccation-tolerant organisms contribute to ecosystem stability and resilience?
- How are global changes in temperature and precipitation impacting the performance and survival of desiccation tolerant organisms?

**Physiological and cellular mechanisms**

- What cellular and molecular processes enable desiccation tolerance in different species?
- How do protective proteins (e.g., LEAs, HSPs, ELIPs) and sugars (e.g., trehalose, sucrose, raffinose) interact to modulate the vitrified properties of a cell and maintain cellular integrity during desiccation?
- What mechanisms restore cellular functions and repair damage upon rehydration?
- How is gene expression regulated during desiccation and rehydration, and what roles do chromatin modifications, non-coding RNAs, and post-transcriptional modifications play?
- How can omics data be integrated to elucidate systems-level mechanisms of desiccation tolerance?

**Data gaps and methodology**

- How can taxonomic sampling of desiccation tolerant specimens be improved?
- How can existing techniques for studying the desiccated state be improved?
- What frameworks are needed to ensure that desiccation tolerance research is inclusive and FAIR (Findable, Accessible, Interoperable, and Reusable)?

**Translational applications**

- How can desiccation tolerance research inspire novel materials and biotechnological innovations?
- How can xeropreservation of complex biological specimens be achieved?
- Can we engineer desiccation tolerance in seeds that are sensitive to desiccation?
- How can principles of desiccation tolerance be applied to enhance plant resilience under water-limiting conditions?

### How do you know if a specimen is desiccation-tolerant?

**Fig. 2:**
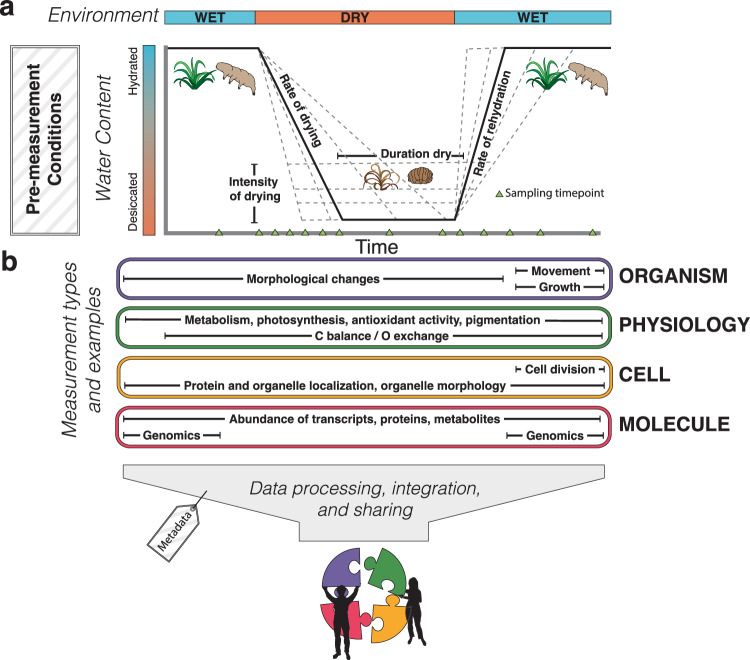
Generalized diagram depicting a desiccation-rehydration time course for representative desiccation-tolerant organisms. **a** Shows organismal water content as a function of environmental water availability and the drying parameters that can be varied (rate of drying, intensity of drying, time in the dry state and rate of rehydration). Boxes in **b** show examples of measurements that can be performed in organismal biology, physiology, cell biology, and multi-omics. Pre-measurement conditions and data integration are important additional considerations. Illustrations in (**a**) by Rachel Torrez.

In order to standardize and integrate across disciplines, we need to agree on how to test if an organism, tissue, or cell is desiccation-tolerant. To determine if a specimen is desiccation-tolerant, we must first observe it in a desiccated state, and subsequently observe it recover when rehydrated. However, this is not as simple as it sounds and many factors impact drying and subsequent recovery. Thus, it is important to test a selection of drying and rehydration scenarios before drawing a conclusion (Fig. 2). Different species tolerate different rates and intensities of drying, durations in a dry state, and rates of rehydration^54,92,143^. Each species likely has an optimal drying scenario that maximizes desiccation tolerance and this can be established empirically by implementing sequential drying experiments (Box 3). Considering the habitat and environment where the organism naturally occurs can inform the design of ecologically relevant drying scenarios and rehydration methods. Additional factors such as the temperature, light, and starting condition of the specimen can also have a substantial impact on survival and these should always be reported. For example, exposure to previous dry or wet conditions can influence recovery from desiccation in some species^144,145^ so the experimental and growth conditions of materials should always be reported. Rehydration methods also matter, and while specimens are commonly rehydrated by the addition of liquid water, in some cases, exposure to high humidity prior to the addition of water improves recovery^146^.

Our working definition of desiccation tolerance hinges on measuring the water status of a specimen in its most dehydrated state, and then detecting viability of the sample after rehydration (Box 1). While measuring water potential directly is ideal^147^, for practical reasons, measuring relative water content (RWC), water content (WC as g H_2_O g dwt^-1^), or drying to equilibration with a known relative humidity (RH), may be more feasible (Box 3). Most methods for the direct measurement of water potentials, such as thermocouple psychrometers, dew point sensors, and pressure bombs have practical or technical limitations^148–150^. It is for these reasons that gravimetry-based methods for assessing water status, including RWC and WC, although less comparable across species^151^, have been adopted (Box 3). Physiological markers associated with water status, such as the cessation of respiration, photosynthesis and other metabolic processes are useful indicators for the early stages of dehydration (between −2 to −15 MPa^20^), and the extent of vitrification may be used to help validate the more extreme changes in water status. We suggest that measures of both water status and physiology be coupled to improve robustness.

Drying to equilibrium with known relative humidities (Box 3) is the most precise method for varying the rate and intensity of desiccation. This approach works well in many organisms such as bacteria, yeasts, algae, bryophytes, and seeds, but is less useful for vascular plants as it does not mimic their natural processes. Desiccation-tolerant animals are typically desiccated by extended exposure to low humidity environments, but it is unclear if drying continues to equilibrium as water status is typically reported as WC^25^. Desiccation-tolerant seeds are routinely dried to equilibrium with air at 10 to 20% RH^152^. However, simply drying an organism to equilibrium with a particular RH to establish if the organism is desiccation-tolerant does not consider the impact of drying rate and rehydration on the tolerance, so again, multiple drying and rehydration scenarios should be assayed.

Our definition of desiccation tolerance also relies on detecting viability after rehydration. Viability is determined by evidence of cellular function after rehydration including metabolism, growth, and reproduction. In practice, this can take multiple forms. Some studies measure growth and development following rehydration, while others measure physiological markers of metabolism and photosynthesis, and others measure water status. Water status alone is not sufficient to determine recovery, and we recommend that a combination of these metrics be reported, with the caveat that researchers measuring growth must distinguish between recovery of existing cells vs. new growth, as only the former demonstrates tolerance. Viability after desiccation is also influenced by the rehydration process, which can impose additional stresses on organisms, so again multiple scenarios should be assayed.

Box 3 Methods of measuring and controlling drying **Water Content (WC):** WC is commonly used to assess the water content of bacteria, yeasts, algae, seeds, roots, and bryophytes and is calculated as WC = (Fwt-Dwt)/Dwt where fresh weight (Fwt) is the initial weight in grams of the specimen and the dry weight (Dwt) is the weight of the specimen in grams after oven drying to constant weight (for vegetative tissues, 65 °C to 75 °C; for seeds, 103 °C as recommended by the International Seed Testing Association (ISTA, 2023)). Care must be taken to avoid the loss of volatiles in vegetative tissues and/or an increase in mass of the dried samples due to absorption of water vapor between removal from a drying oven and weighing. Drying to 0.1 g H_2_O g dwt^-1^ is reported to be roughly equivalent to drying to −100 MPa^200^ and would thus serve as a determinant for classification for desiccation tolerance based on our working definition. WC assessments can be made more rigorous by verifying that tissues have stabilized and are no longer decreasing in mass despite continued exposure to a constant dry environment, suggesting equilibration.

**Relative Water Content (RWC):** RWC measures the water content of a tissue relative to the weight of the tissue at full turgor, and is calculated using the equation: RWC = (Fwt-Dwt)/(FTwt-Dwt) × 100 where Fwt = Fresh weight, Dwt = Dry Weight and FTwt = Fresh weight at full turgor^201^. Once the fresh weight of the tissue has been recorded the full turgor weight is obtained by submerging the sample in water until the sample has reached a constant weight (timing must be determined empirically for each tissue type). In some cases, the tissue can be floated on the surface or, in the case of some leaves, by submerging the specimen in water. The establishment of the full turgor weight should include removal of visible external water and be accomplished at 4 °C and in the dark to minimize the impact of respiratory and/or photosynthetic activity^201^. The tissue dry weight is determined by oven drying the sample at 65 °C to 75 °C for 24 to 48 h. Although this is a useful measure in plant based studies, there is no direct way of relating RWC to the water potential of the tissue. However, RWC can be calculated from WC/WC_F_ where WC_F_ is the specific water content at full turgor. Drying to 10% RWC is roughly equivalent to −100 MPa. RWC also comes with a caveat in that it is not a very sensitive indicator of plant water status when the water deficit is not severe^202^.

**Equilibrium drying:** Equilibrium drying is achieved by placing samples in a closed chamber over a saturated salt solution that maintains a stable RH at a given and constant temperature, and then allowing sufficient time for the samples to reach a constant weight. The specific RH within the chamber is determined by the physico-chemical properties of the salt and can range from 98 to 8% depending upon the temperature^151^ Once the samples maintain a constant weight, the water potential of the organisms or tissues are at equilibrium with the water potential of the surrounding air. The water potential of the air, and by extension the water potential of the sample at equilibrium, can be calculated using the formula Ψ = (*RT/V*_*w*_) *ln* (%*RH/100*) where Ψ = water potential, *R* = the gas constant, *T* = temperature in degrees Kelvin, and *Vw* = the partial molar volume of water^151^. Water activity (a_w_) is also often used to describe the water content of a tissue, and involves equilibrium drying such that at the equilibrium RH (the sample would be at equilibrium) ***RH*** ***=*** ***a***_***w***_ ***X 100***. For an organism or tissue to be considered desiccation-tolerant, it must be equilibrated with a combination of RH and temperature that would deliver a water potential of −100 MPa or less (an a_w_ value of 0.5 or below).

**Sequential drying experiments:** Sequential exposure to a descending series of RHs can be used to manipulate the rate and intensity of drying. Specimens can be removed and rehydrated at different points along the sequential drying process. In addition, specimens can be maintained at intermediate RHs for differing amounts of time to modify drying rate. Drying rate can also be slowed down by use of a wetted substrate at a single RH (preferably 50%) to slow the water loss^203^; the latter forms a region of high humidity at the surface of the substrate that slowly dissipates with time^55^.

**Measuring viability:** Viability can be determined by screening for cell division, tissue growth, organismal movement, or physiological markers. In the context of desiccation tolerance, it is critical that viability be assessed in cells that underwent desiccation or their daughter cells. Cell division and growth are simple metrics and can be used in most systems. Cell division can be measured by using a compound microscope, in microbes by testing for colony forming units^165^, or by visible tissue growth in plants^90^. In some organisms like *C. elegans* and tardigrades, coordinated organismal movement after desiccation^134,204^ may be a more appropriate metric than cell growth or division because these organisms only undergo cell division in some stages of their lifecycle. In early stages of recovery, changes in water content can serve as a proxy for viability using infrared thermography. This technique has been used to visualize and measure RH-controlled “thermal fingerprints” of seeds and several species of desiccation-tolerant lichens^205^, though this may pose issues in organisms that take up water when they are dead. In light of this, physiological markers associated with water status such as the resumption of metabolism and photosynthesis, should be used to help determine viability after rehydration.

### How do you measure physiological and cellular responses to dehydration in desiccation-tolerant organisms?

A suite of physiological processes linked to desiccation tolerance can be measured to gain insight into the degree of stress and mechanisms of tolerance. Most of these manifest in the early stages of desiccation, while water still remains in the tissues. The majority of cellular processes cease at water potentials below −15 MPa^20,153^ and the components required for desiccation tolerance likely need to be assembled prior to this. Cellular processes vary in their need for water: protein and nucleic acid synthesis ceases when water potentials reach approximately −5 MPa, respiration ceases at ~ −15 MPa, and enzyme activity ceases at ~ −25 MPa^153^. Biochemical changes at water potentials below this are likely determined by chemical activity and physical forces and are not biologically driven. The specific water requirements for these processes vary across life forms and tissues, highlighting the need for careful characterization of each desiccation-tolerant organism under consideration. Measuring these parameters across a drying timecourse can help to determine the water content at which various cellular processes cease.

Many properties and pathways involved in desiccation tolerance can be surveyed across drying time courses, including oxidant and antioxidant activity, changes in pigment concentration, and in the case of plants, chlorophyll fluorescence parameters such as the maximum (*F*_*V*_/*F*_*M*_) or effective (ΦPSII) quantum yield of photosystem II. To complement these traditional physiological measures, changes in the amount and combination of various sugars, such as trehalose, sucrose, raffinose family oligosaccharides, and other metabolites can be tested via metabolomics^135^, and proteins and transcripts can be quantified via proteomics and transcriptomics. For desiccation-tolerant organisms that undergo dry-wet-dry cycles in nature, the carbon balance metric^82,154,155^, employing infrared gas analysis, is especially illustrative of physiological tolerance. While this technique was originally developed for use with photosynthetic organisms, any organism that exchanges gas with a headspace (e.g., biocrust communities^156^) can be measured with respect to carbon balance, providing insights into the relative level of metabolism during dry-wet-dry cycles. Carbon balance measurements can be applied to communities to assess combined and emergent phenotypes or on individual community members (excised or grown separately) to examine individual roles of organisms^82^. Similarly, oxygen consumption can be measured in non-photosynthetic anhydrobiotes (e.g., brine shrimp, nematodes, tardigrades) during desiccation^157^. Biophysical and material properties of dried organisms, like changes in cytoplasmic vitrification, stability of sugar glasses, molecular mobility, H-bonding patterns, and molecular packing are informative and can be assayed by various forms of spectroscopy^158,159^. Traditionally, glass transition temperature has been considered a key protective property distinguishing desiccation-tolerant from sensitive vitrified systems. However, recent work suggests that properties such as glass fragility can be just as important in conferring desiccation tolerance and therefore warrant further research attention^139^.

Viewing organisms and cells in hydrated, dehydrated, and rehydrating states is useful and relies on microscopy and, in some cases, spectroscopy. Changes to the structure of cells during desiccation and rehydration can be observed using electron and light microscopy^160^ if appropriate fixation techniques are utilized to prevent rehydration of dried samples. To observe changes in protein localization and organelle morphology during desiccation and recovery, proteins can be tagged with fluorescent markers and visualized by fluorescent microscopy^122,125,161^. Changes in cellular rehydration can be measured using genetically encoded multimeric nanoparticles^162^, which are fluorescently labeled particles within cells that allow monitoring of changes in sub-cellular diffusion and cytoplasmic viscosity^163^. Similarly, changes to intracellular molecular crowding during osmotic stress can further be assayed using the Förster Resonance Energy Transfer based sensor SED1^164^. Cell viability and division can be measured either by using appropriate stains, microscopy, or growth assays such as colony forming units in microbes^165^, or tissue growth in plants^90^. In addition, cell membrane phase behavior can be quantified using Fourier transform infrared spectroscopy^166^, and the viscosity of tissue can be probed by electron paramagnetic resonance^158^.

### How do you integrate omics data to understand desiccation tolerance at the systems level?

Systems-level analyses coupling genomics, transcriptomics, proteomics, and metabolomics with traditional physiology and cell biology offer powerful tools to elucidate the complex mechanisms underlying desiccation tolerance. Desiccating cells undergo massive shifts in transcriptomic, proteomic, and metabolomic profiles, and capturing these changes has been central to dissecting the mechanisms of desiccation tolerance. However, these high-dimensional omics studies often suffer from inconsistent methodologies and incomplete metadata reporting, similar to those observed more broadly in the plant drought response literature^167^. It is also important to recognize that the presence of a compound (e.g., mRNA, protein, metabolite) does not provide information about the balance between synthesis and degradation. Indeed, there is often very little correlation between transcript abundance and protein synthesis^168^, and this also extends to the relationship between protein abundance and metabolite levels^169^. As cells dry, their ability to synthesize transcripts, proteins, and metabolites becomes compromised^20^. Thus, increases in transcripts, proteins, and metabolites late in the drying process are likely the result of changes in turnover rates, stability, or sequestration, as seen in dehydrating *Syntrichia ruralis*^170^. This is particularly relevant to studies that are focused on the longevity of desiccated samples where alterations in cellular components (transcripts, metabolites, etc.) are likely to result from instability, chemical activity, or physical forces rather than biological activity since cellular processes are non-operative in the desiccated state. Such samples may also contain changing fractions of potentially viable and nonviable components (cells, tissues), which can be difficult to decipher in pooled samples. This begs the question as to whether or not components that accumulate late in the drying process are important during drying, are necessary for recovery upon rehydration, or are needed to replenish depleted pools.

Paired multi-omic datasets with complete metadata are needed to decipher these dynamics (Fig. 2). Recent technological advances that enable measurements of genome accessibility and ribosome occupancy can help resolve these uncertainties and many types of measurements can be performed simultaneously^171^. Experiments that are designed to integrate physiological, cellular, and -omic level processes will allow for critical connections between form and function in desiccation tolerance research. Expanding these studies further to investigate community level responses is another important area for future research. Such experiments also provide an obvious opportunity to collaborate across labs and institutions to bring diverse expertises together.

### How do you manage, curate, and share your desiccation tolerance research data?

Desiccation tolerance research has generated large volumes of data across all omics modalities as well as established and emerging laboratory and in silico techniques. Such data can be expensive to generate and, in the absence of harmonized metadata referring to common technical standards, difficult to integrate between experiments, let alone between laboratories^167^ and sub-disciplines.

To make efficient use of research funds and embrace productivity gains from emerging computational techniques, data must be shared in a FAIR (Findable, Accessible, Interoperable and Reusable) manner. An attempt to achieve this has been initiated for desiccation-tolerant plants in the Drying without Dying database^62^, but community buy-in and extension to additional study organisms are needed. Shared practices should not only apply to the laboratory, but be documented and annotated with appropriate machine and human-readable metadata (Supplementary Table 1), and deposited in reputable repositories that can guarantee longevity (Supplementary Table 2). Appropriate metadata standards should capture and report sample histories and status, and relevant environmental parameters such as the RH, light, temperature, and water status. Standardized physiological data (e.g., water status, respiration, photosynthesis, etc.) should accompany all omics studies to enable comparative analyses, and sampling times should account for circadian regulation. Relevant standards and guidelines for reporting data and metadata are listed in Supplementary Table 3.

## Prospects and future vision

**Fig. 3:**
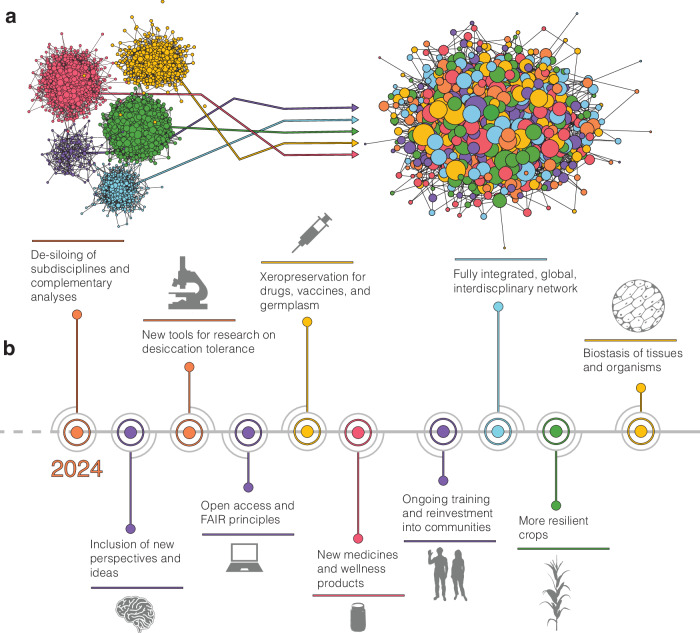
Desiccation tolerance research, going forward. **a** De-siloing sub-disciplines of desiccation tolerance research. **b** Future prospects and possible applications of desiccation tolerance research. In (**a**), colors represent research in desiccation tolerance at different biological scales (evolution, geography, organism, cell, and molecule). In (**b**), colors represent broad categories for aspirational goals in desiccation tolerance research: purple for social and community growth and development, blue for geographical advancement, green for organismal-scale applications, yellow for cellular-scale applications, and red for applications on the molecular scale. Orange represents broad, general advancements in desiccation tolerance research.

Research on desiccation tolerance has immense potential (Fig. 3). The adoption of standardized methods and FAIR data practices will streamline the development of novel medical drugs and biofluids, enhance techniques for the dry storage of cells, and stable biologicals such as therapeutic antibodies and mRNA vaccines, and provide genetic tools for engineering biostasis. This standardization could also accelerate the engineering of crops that are more resilient to water limitation, improve seed priming and conservation efforts, and target pathogen dormancy, while inspiring the creation of new environmentally responsive materials that mimic these biological processes for industrial, agricultural, and medical applications.

The study of desiccation tolerance has long promised to improve human health and medical technology. For example, understanding how pathogens or disease vectors tolerate desiccation will help combat the spread of disease and could yield new drug targets^172–174^. Metabolites such as trehalose, phenolic compounds, and essential oils derived from desiccation-tolerant organisms have been recognized for their antioxidant, anti-obesity, anti-inflammatory, antimicrobial, osmotic stress reducing, and chemoprotective properties, and thus carry potential for cosmetics, nutrition, and food storage^175^. Extracts from several desiccation-tolerant plants are already used in anti-aging, skin protective, and moisturizing lotions. Dietary intake of these compounds has been associated with lower rates of cancer, diabetes, and cardiovascular diseases and might improve meat quality, growth, and gut function when used in animal nutrition^176–182^. However there is a lack of information on possible side effects or contaminations in these extracts and low biomass production challenges the sustainable harvesting of desiccation-tolerant organisms. These challenges should be tackled by the development of effective production systems, regulations to ensure sustainable use, and comprehensive toxicological studies, guidelines, and regulatory frameworks to facilitate safe consumer products^178,182–184^.

Promising applications also exist in “xero-” or dry-preservation of biological materials that would mitigate the dependence on costly and logistically difficult cryo-preservation and cold chain logistics. Currently, the state of the art lies in xeropreservation of simple biologics such as vaccines and other molecular therapeutics^185,186^ as well as cell-based biologics including platelets and stem cells^187–190^, but xeropreservation of tissues and increasingly complex systems could be on the horizon. Advancing these techniques will require refinement of preservation techniques, possibly leveraging novel excipients, formulations, and loading methods. Future advances could someday move beyond molecules and cells to tissues, organs, or even whole-organism stabilization. Of course, immunogenicity and toxicity studies are needed to assess the safety of novel excipients and biologics stored under new conditions, but this area holds promise.

Desiccation-tolerant plants and seeds also hold a wealth of information relevant to agriculture, biotechnology, and material science. Through direct genetic manipulation of key pathways and their regulators, desiccation-sensitive seeds and crops could be made more tolerant of water deprivation. Seeds of many tropical plants, including high-value crops such as coffee, cocoa, and mango are desiccation sensitive. Understanding desiccation sensitivity and tolerance in these seeds is crucial for safeguarding the germplasm of this important biodiversity. Even for traditional crops which are unlikely to face vegetative desiccation, research on desiccation tolerance mechanisms could help improve performance under periodic water deficits. For instance, desiccation inspired traits related to osmotic adjustment, protective pigments, and oxidative stress management could inform breeding strategies that enhance recovery under drought stress. We acknowledge that extreme water deficit experienced by desiccation-tolerant organisms differs substantially from milder, sub-lethal drought stresses that agricultural crops are faced with. However, metabolic and protective pathways activated in desiccation-tolerant plants could be used to enhance survival across a spectrum of water-limited conditions, especially in subsistence agriculture, not just for engineering crops that survive desiccation outright. As modern crop breeding shifts focus from purely maximizing productivity to enhancing resilience and ecological functionality, desiccation tolerance research provides an opportunity for developing crops that balance productivity with resilience under marginal conditions. Studies have already demonstrated that overexpression of LEA, HSPs, and other key desiccation tolerance genes can improve photosynthetic rates and recovery under moderate drought stress^191–194^. While industrialized, high-input agriculture seeks to optimize yield, in regions where drought-related crop failures can lead to total food insecurity (e.g., smallholder or subsistence farming systems), having a crop that can survive a period of water deficit—enabling partial yield after rain returns—would be transformative. Indirect approaches, which circumvent the negative public perception of genetically modified organisms and the plant transformation bottleneck, should also be exploited. For example, desiccation-tolerant plants harbor valuable microbial diversity that can be leveraged to enhance microbe-mediated drought tolerance^195–197^. The root-associated microbiota of multiple desiccation-tolerant plants are currently being tested as biostimulants and stress-tolerance enhancers with promising results^198,199^.

### Maximizing potential through interdisciplinary collaboration and community building

Tremendous progress in understanding desiccation tolerance has been driven by diverse research communities around the world. These communities have pushed our understanding of how organisms across the domains of life survive desiccation, providing insight into the biophysical, molecular, cellular, organismal, ecological, and evolutionary mechanisms of tolerance. With these advances, our hope is that we are now on the verge of unifying the field to translate these results to practical uses.

In order to achieve these lofty goals, global partnerships and interdisciplinary collaborations are needed. Collaborations that span the scientific disciplines of biology, engineering, computer science, and beyond should be coupled with partnerships across governmental agencies, entrepreneurs, and local communities. Integrating expertise and principles from material engineering, computer science, and biophysics will accelerate the development of new tools for studying biology in a dry state and analytical approaches for integrating multidimensional datasets. Of course, partnerships with governmental agencies, non-profit organizations, and entrepreneurs are critical for bringing advances to consumers while ensuring legal and ethical compliance.

Breaking down the silos within desiccation tolerance research will require investing in collaborations across diverse disciplines and global regions. Given that desiccation-tolerant organisms are widely distributed with many important diversity hotspots in the Global South, collaborations should respect the sovereignty and intellectual property rights of local and indigenous communities, with equitable benefit-sharing agreements established at their inception to ensure compensation for germplasm or traditional knowledge. Partnerships should also ensure that technologies for studying desiccation tolerance are both accessible and affordable. Open access to research tools, data, and sharing platforms can democratize science, allowing a broader range of researchers, including those from less affluent regions, to participate. We advocate for developing methods and approaches that are cost-effective and easily accessible across different geographical and economic landscapes. By continuing to encourage and push for an inclusive, equitable, and interdisciplinary global research network, we can help support a new standard for desiccation tolerance research.

## Supplementary information

Supplementary Information
