# Supplementary material for: Life on the dry side: a roadmap to understanding desiccation tolerance and accelerating translational applications
Source: Nat Commun. 2025 Apr 6;16:3284. doi: 10.1038/s41467-025-58656-y (PMC11973199; doi:10.1038/s41467-025-58656-y)
Supplement: Supplementary file 1 — Supplementary Information [file 41467_2025_58656_MOESM1_ESM.pdf]

Supplementary Information

Life on the dry side: A roadmap to understanding desiccation tolerance and accelerating translational applications

Authors: R. A. Marks, J.T.B. Ekwealor, M.A.S. Artur., L. Bondi, T.C. Boothby, O.M.S. Carmo, D.C. Centeno, K.K. Coe, H.J.W. Dace, S. Field, A. Hutt, S. Porembski, A. Thalhammer, L. van der Pas, A.J. Wood, P. Alpert, D. Bartels, S. Boeynaems, M.N. Datar, T. Giese, W.I. Seidou, S.M. Kirchner, J. Köhler, U.G.V.S.S. Kumara, J. Kyung, R. Lyall, B.D. Mishler, J.B.V.T. Ndongmo, M.S. Otegui, V. Reddy, J. Rexroth, S.M. Tebele, R. VanBuren, J. Verdier, U.C. Vothknecht, M.F. Wittenberg, E. Zokov, M.J. Oliver, S.Y. Rhee

Table S1: Essential organismal metadata

**Category**	**Variable**
Study organism	species, cell line, accession number (if applicable), ecotype, genotype, sex
Growth conditions	field collections: location, season, habitat description; lab conditions: temperature, relative humidity, vapor pressure deficit, light, soil, growth media
Sampling	tissue type, age, developmental stage, timepoint, water status, relevant physiology (e.g., photosynthesis, metabolism), replicate number, mass
Verification of desiccation recovery or survival	resumption of metabolism, photosynthesis, or growth and reproduction

Table S2: Databases for open access and interoperable data storage

**Datatype**	**Database URL**	**Notes**
Microscopy	https://www.ebi.ac.uk/bioimage-archive/ https://www.ebi.ac.uk/empiar/	Bioimage archive for light microscopy images; Electron microscopy data bank for electron micrograph datasets
Proteomics	https://www.ebi.ac.uk/pride/	Proteomics identification database
Metabolomics	https://www.ebi.ac.uk/metabolighhttps://www.ebi.ac.uk/metabolights/	MetaboLights recommended

	ts/; http://www.metabolomexchange.org/site/ https://gnps.ucsd.edu/ProteoSAFe/static/gnps-splash.jsp	for data submission; Metabolights and other repositories searchable at MetabolomeXchange; GPNS for mass spectrometry data
Sequencing	https://www.ncbi.nlm.nih.gov/sra	Sequence read archive
Assembled genomes	https://ncbi.nlm.nih.gov/genome	Genome archive
All data	https://zenodo.org/	Repository for all datatypes, useful when more specialized data repositories do not exist but not ideal for findability or interoperability

Table S3: Relevant standards and guidelines for reporting data and metadata

**Reporting guidelines topic**	**URL**	**Notes**
Plant phenotyping experiments	https://www.miappe.org/	Suitable for phenotyping experiments of non-plant anhydrobiotes as well
Image acquisition, analysis, and publishing standards	https://doi.org/10.1038/s41592-023-01987-9	Community-developed checklists for publishing images and image analyses
Proteomics	https://www.psidev.info/miape	Minimum information about proteomics experiments
Reporting guidelines for mass spectrometry-based metabolomics	https://doi.org/10.1038/s41592-021-01197-1	Includes guidance for annotation and quantification
Reporting guidelines for bulk RNA sequencing	https://www.encodeproject.org/data-standards/rna-seq/long-rnas/	Data standards and processing pipeline
Reporting guidelines for assembled genome submission	https://www.ncbi.nlm.nih.gov/genbank/genomesubmit/	Creation of a BioProject or BioSample is required prior to genome submission.
